# First Report of *Scutellonema brachyurus* (Steiner, 1938) Andrassy, 1958 and Occurrence of *Meloidogyne incognita* (Kofoid & White, 1919) Chitwood, 1949 in Belgium

**DOI:** 10.21307/jofnem-2019-062

**Published:** 2019-09-17

**Authors:** Huu Tien Nguyen, Quang Phap Trinh, Marjolein Couvreur, Phougeishangbam Rolish Singh, Wilfrida Decraemer, Wim Bert

**Affiliations:** 1 Institute of Ecology and Biological Resource, Vietnam Academy of Sciences and Technology, 18 Hoang Quoc Viet, Cau Giay, Hanoi, Vietnam; 2 Graduate University of Science and Technology, Vietnam Academy of Sciences and Technology, 18 Hoang Quoc Viet, Cau Giay, Hanoi, Vietnam; 3 Nematology Research Unit, Department of Biology, Ghent University, K.L. Ledeganckstraat 35, 9000 Ghent, Belgium

**Keywords:** Global warming, *Hedychium greenii*, *Musa basjoo*, mtDNA, *Nad5*

## Abstract

A study of plant-parasitic nematodes in the Botanical garden at Ghent University in Belgium revealed the presence of two tropical nematode species, i.e. *Scutellonema brachyurus* and *Meloidogyne incognita*. *Scutellonema brachyurus* was recovered, only once, for the first time in Belgium from *Musa basjoo* and is morphologically characterized. *M. incognita*, forming galls on *Hedychium greenii*, was recovered in all seasons over three consecutive years and is morphologically and molecularly characterized. Although no unequivocal evidence was found to indicate that these nematodes pose a current threat in Belgium, in the light of climate change, it is crucial to improve our knowledge of potential tropical nematode activity in more Northern countries.

Recently, the presence of tropical nematodes has been reported from several Mediterranean or more southern European countries (Wesemael et al., 2011; EPPO, 2019). However, tropical nematodes do not yet pose a problem in the more northern parts of Europe, including Belgium. According to Bebber et al. (2013), there is clear evidence of a general, climate change-driven, poleward migration of pests and plant pathogens, a movement that can include tropical plant-parasitic nematodes. Interestingly, our survey revealed the presence of *Scutellonema brachyurus* and the tropical root-knot nematode *Meloidogyne incognita* (Kofoid and White, 1919) Chitwood, 1949 for the first time, respectively, on banana (*Musa basjoo* Siebold & Zucc. ex Iinuma) and red ginger (*Hedychium greenii* W. W. Sm.) in Belgium.

## Materials and methods

After removing detritus layer from the surface, soil and root samples were collected from the upper 30 cm soil layer around the rhizosphere of *Musa basjoo* and *Hedychium greenii* at the Botanical garden of Ghent University (GPS coordinates: N: 51°2′6.8″, E: 3°43′22.7″ and N: 51°2′6.7″, E: 3°43′22.4″, respectively). Several samples were taken from September 2017 to June 2019 to check for the survival of nematodes through the winter season. For morphological characterization, vermiform nematodes were extracted by the modified Baermann tray method (Whitehead and Hemming, 1965). After that, permanent slides were made following Singh et al. (2018). Mature females of *M. incognita* were extracted directly from the galls under a stereomicroscope, using a scalpel and forceps. Perineal patterns were cut and cleaned following Hartman and Sasser (1985) and mounted in glycerine. Microphotographs were made from permanent slides using an Olympus BX51 DIC Microscope equipped with a digital camera. Measurements were made based on the obtained pictures using Image J 1.51 (Schneider et al., 2012). For molecular characterization, primers NAD5F2/NAD5R1 were used to amplify the *Nad5* mtDNA gene following the protocol of Janssen et al. (2016). *Nad5* mtDNA sequences of *M. incognita* in Belgium were aligned with 73 reference sequences of tropical root-knot nematode species from Janssen et al. (2016) and other closely related sequences from GenBank using Muscle on Geneious R11 (www.geneious.com) to check for polymorphic nucleotide positions.

## Results

### Scutellonema brachyurus (Steiner, 1938) Andrássy, 1958

(Fig. 1, Table 1).

**Table 1.** Measurements of *Scutellonema brachyurus* and *Meloidogyne incognita* from Belgium

	S. brachyurus (Belgium)	M. incognita (Belgium)		
	Females	Juveniles	Males	Females
*n*	11	20	10	10
Body length (L)	699 ± 50 (625–774)	406 ± 17.1 (374–420)	1884.0 ± 135 (1728–2048)	584 ± 66.8 (506–751)
*a* = L/MBD	28.3 ± 7 (23.8–44)	27.4 ± 2.4 (23.5–32.6)	48.5 ± 4.0 (44.9–54.2)	1.8 ± 0.1 (1.5–2.0)
*b* = L/anterior to pharyngo-intestinal valve	6.6 ± 0.44 (5.9–7.2)	–	–	–
*b*' = L/ anterior to base of pharyngeal gland	5.5 ± 0.42 (4.9–6.1)	3.0 ± 0.3 (2.4–3.5)	7.9 ± 0.6 (7.3–8.6)	–
*c* = L/Tail length	79 ± 44 (49–177)	8.4 ± 0.4 (7.7–9.0)	204.4 ± 107.6 (137.1–365.1)	–
*c*' = Tail length/ABD	0.66 ± .06 (0.55–0.76)	5.0 ± 0.4 (4.5–5.6)	0.4 ± 0.3 (0.1–0.6)	–
V%	59 ± 1.5 (57–62)	–	–	–
Distance from anterior end to middle of genital primordium	–	254 ± 18.2 (228–279)	–	–
Lip height	5.7 ± 0.4 (5.1–6.2)	–	7.2 ± 1.1 (6.3–8.8)	–
Lip width	9.1 ± 0.37 (8.7–9.6)	–	13.4 ± 0.3 (13.2–13.9)	–
Stylet length	26.8 ± 1.1 (25.2–28.6)	10.9 ± 0.8 (9.5–12.0)	24.7 ± 0.9 (23.9–25.8)	15.6 ± 0.7 (14–17)
Conus length	14.1 ± 1.1 (12.7–15.8)	6 ± 0.5 (5–6)	13.9 ± 0.5 (13.2–14.5)	9 ± 0.9 (8–10)
Shaft length	10.1 ± 0.39 (9.5–10.5)	4 ± 0.6 (3–5)	8.0 ± 0.6 (7.6–8.8)	4.8 ± 0.7 (4–6)
Knob height	2.7 ± 0.39 (2.3–3.4)	1 ± 0.0 (1–1)	2.8 ± 0.4 (2.5–3.2)	2.3 ± 0.3 (2–3)
*m* = Cone/Stylet	0.52 ± 0.03 (0.48–0.57)	–	–	–
*o* = DGO*100/Stylet	19.4 ± 2.0 (17.1–22.9)	–	–	–
Distance from dorsal gland orifice to stylet base	5.2 ± 0.53 (4.6–6.0)	4 ± 0.5 (3–4)	3.8 ± 0.7 (3.2–4.4)	3.8 ± 0.6 (3–4)
Anterior end to secretory-excretory pore	116 ± 5.2 (110–124)	84 ± 3.4 (79–89)	176.4 ± 14.2 (156.2–189.6)	23 ± 7.8 (16–38)
Anterior end to nerve ring	84 ± 1.5 (82–86)	71 ± 3.4 (66–77)	126.9 ± 12.0 (110.3–138.6)	–
Anterior end to end of pharyngeal gland	127 ± 4 (120–134)	139 ± 15.5 (117–173)	239.7 ± 2.2 (237.5–241.9)	–
Pharyngeal gland overlapping	21.9 ± 3.9 (15–26.9)	–	–	–
Anterior genital tract	178.9 ± 16.6 (168–203)	–	–	–
Posterior genital tract	173 ± 5.7 (168–181)	–	–	–
Diam. at mid-body (MBD)	27.1 ± 2.4 (23.8–31)	15 ± 1.5 (13–18)	38.9 ± 2.5 (35.9–41.0)	327 ± 55.0 (259–430)
Diam. at anus (ABD)	17 ± 0.89 (16.2–18.3)	10 ± 0.6 (9–11)	71.7 ± 101.3 (18.9–223.7)	–
Tail length	11.2 ± 1.2 (9.8–13.3)	50 ± 2.5 (46–53)	10.7 ± 3.8 (5.0–13.2)	–
Hyaline length	4.2 ± 0.51 (3.7–5.0)	15 ± 2.1 (13–20)	–	–
Spicule length (along arc)	–	–	33.5 ± 5.1 (28.4–40.3)	–
Maximum spicule width	–	–	4.1 ± 0.6 (3.8–5.0)	–
Gubernaculum length	–	–	11.0 ± 1.2 (10.1–12.6)	–
Number of tail annuli at ventral side	9.4 ± 0.54 (9–10)	–	–	–
Scutellum diameter	3.4 ± 0.28 (3.1–3.8)	–	–	–
Length of cervical region	–	–	–	243 ± 37.9 (184–311)
Anterior end to end of metacorpus	–	–	–	81 ± 10.5 (67–101)
Metacorpus diameter	–	–	–	36 ± 6.4 (28–47)
Vulva slit length	–	–	–	18.7 ± 2.9 (15–23)
Vulva width	–	–	–	23 ± 3.2 (19–28)
Vulva-anus distance	–	–	–	16.2 ± 2.2 (12–18)

**Figure 1:**
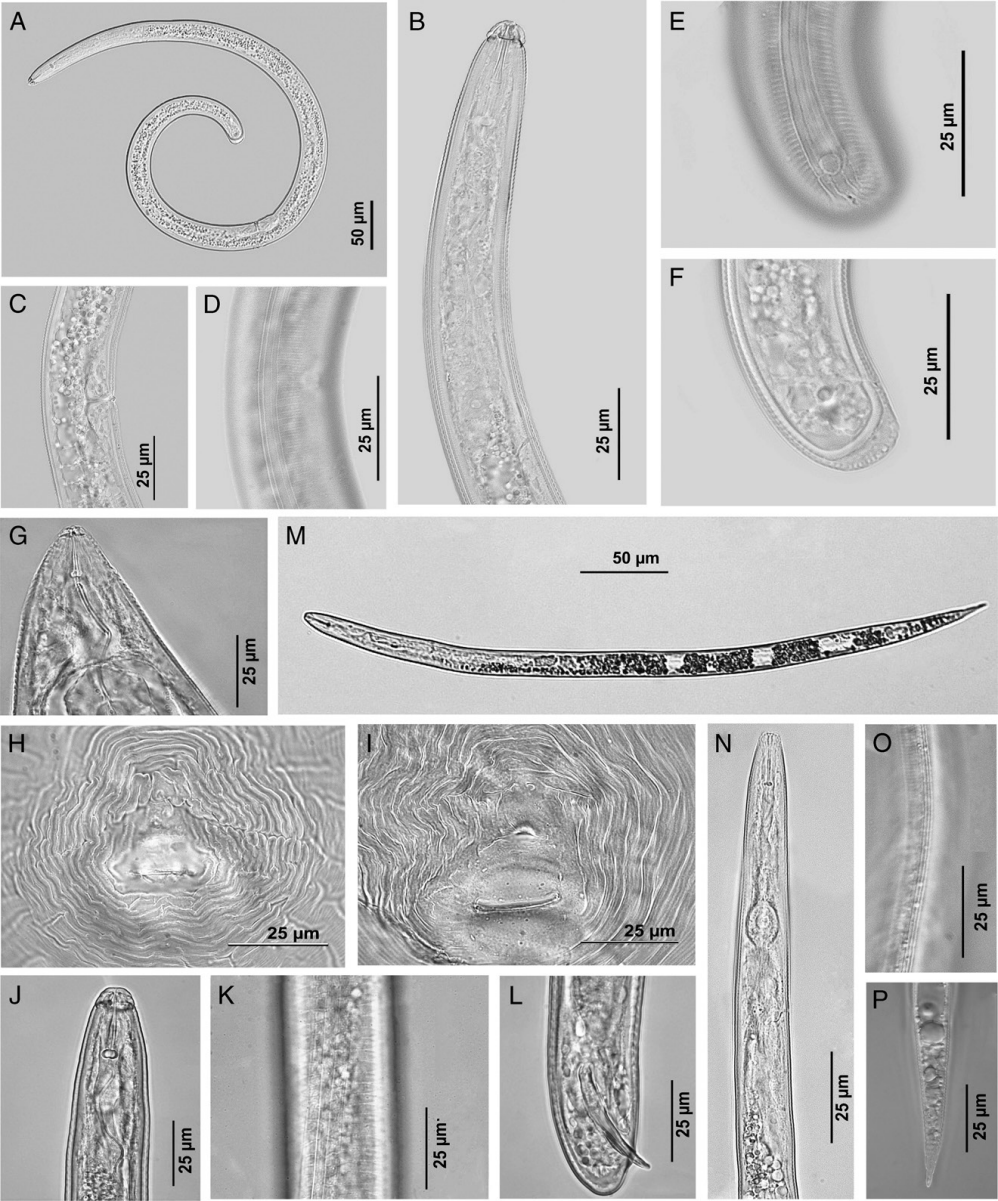
LM pictures. (A–F) females of *Scutellonema brachyurus* from Belgium. (A) Entire body; (B) Anterior region; (C) Vulva region; (D) Lateral field at vulva region; (E) Scutellum at tail region; (F) Tail region. (G–P) *Meloidogyne incognita* from Belgium. (G–I) Mature females. (G) Anterior region; (H, I) Perineal patterns. (J–L) Males. (J) Anterior region; (K) Lateral field; (L) Tail region showing copulatory apparatus. (M–P) Second-stage juveniles. (M) Entire body; (N) Anterior region; (O) Lateral field; (P) Tail region.

#### Remarks

Morphological traits and measurements of the female Belgian population of *S. brachyurus* is in full agreement with the type population of Steiner (1938), except for the slightly shorter stylet (26.8±1.1 (25.2-28.6) µm *vs* 29 µm). However, this variation is known in other populations of *S. brachyurus* (Table 1). Van Den Berg et al. (2013) described two types of *S. brachyurus* (type A and type B), and *S. brachyurus* type A can be differentiated from type B by the main following traits: lip region with 4 to 6 annuli *vs* mainly three, rarely 4 to 5 annuli; 4 to 12 blocks on basal annulus *vs* 8 to 20 blocks; secretory–excretory pore located opposite anterior part to mid-region of overlapping pharyngeal lobe *vs* from rarely opposite mid-isthmus to mostly opposite the posterior part of pharyngeal gland lobe up to its posterior border. The Belgian population of *S. brachyurus* belongs to *S. brachyurus* type B. Unfortunately, our effort to recover the Belgian population of *S. brachyurus* for molecular analysis was unsuccessful. Males were also not found.

### Meloidogyne incognita (Kofoid and White, 1919) Chitwood, 1949

(Fig. 1, Table 1).

#### Remarks

In general, the morphology of *M. incognita* in Belgium is in agreement with the description of Whitehead (1968) (the perineal pattern of Belgian population of *M. incognita* belongs to *M. incognita incognita* type). Only a few variations of measurements were observed such as a wider upper range of body length of juveniles (406 (374-420) µm *vs* 371 (337-403) µm) and a larger DGO value of the males (3.8 (3.2-4.4) *vs* 2.1 (1.4-2.5)). However, these variations are small and fall within the range known from other populations (Table 1). Six *Nad5* sequences were obtained with a length from 544 to 599 nucleotides. The sequences of *M. incognita* in Belgium were identical to each other and five other reference sequences of *M. incognita* (specimen ID: T384, T532, M8, M20, M21) from Morocco, Egypt, and Tanzania in the study of Janssen et al. (2016). During winter time, the aerial parts of the host plant were cut down and the growing area was covered by wood chips. *Meloidogyne incognita* has been found at any time of sampling in all seasons over three consecutive years.

## Discussion

This study reveals the presence of *S. brachyurus* and *M. incognita* in Belgium, species known to be prevalent in warm areas, especially in tropical regions (CABI, 2019). Morphologically, the Belgian population of the spiral nematode belongs to the *S. brachyurus* group type B according to Van Den Berg et al. (2013). However, the presence of cryptic species in the *S. brachyurus* group have only been molecularly unequivocally defined (Van Den Berg et al., 2013; Van Den Berg et al., 2017), and therefore, molecular data are needed to confirm the group of *S. brachyurus* to which the Belgian population belongs. Unfortunately, multiple attempts to recover this nematode for molecular studies failed.

The morphological identification of tropical root-knot nematodes is known to be greatly hampered by phenotypic plasticity and interspecific similarities (Hunt and Handoo, 2009), for example the variation in the number of post-labial annuli of *M. incognita* in Belgium (ranging from 1 to 3 annuli) confirmed the plasticity of the numbers of head annuli in the genus *Meloidogyne*. Therefore, we used an integrated approach including the mitochondrial barcode region *Nad5* as a reliable marker to identify tropical root-knot nematodes.

The Belgian population of *M. incognita* was discovered for the first time on a specimen of *Hedychium greenii* that was planted outside several years ago in the botanical garden of Ghent University, with the accompanying Belgian weather conditions (cold winter seasons). This plant is an exotic plant that was imported from the Himalayas, which in its native habitat can be found growing at altitudes up to 5000 feet (about 1666m), and it is known to tolerate temperatures as low as 15 °F ( −9°C). However, *M. incognita* is known as a tropical nematode and distributed in warmer climates (Wesemael et al., 2011). Most likely, this nematode originated from one of the tropical plants that were imported and planted in the botanical garden of Ghent University. The fact that the growing area was covered by wood chips during winter time may have created more suitable conditions for *M. incognita*.

*Meloidogyne incognita* has already been reported in Belgium by Coolen et al. (1974) in nurseries growing multiflora tuberous begonias. However, without detailed taxonomical information, its species status can therefore not be assured.

Although in this study the presence of *S. brachyurus* and *M. incognita* in Belgium was demonstrated and a climate change-driven poleward migration of pests and plant pathogens is well known (Bebber, 2015; EPPO, 2019), we have insufficient data to state that the tropical nematodes found in this study form a potential threat nor that they are the result of global warming. Nevertheless, in the light of climate change it is highly important to gain more insights in tropical nematodes in more Northern countries. This current study provides the first report of *S. brachyurus* and confirms the presence of *M. incognita* in Belgium, although in a protected environment.
